# Gratitude and Suicide

**DOI:** 10.31486/toj.22.0010

**Published:** 2022

**Authors:** Marianne Maumus

**Affiliations:** Department of Hospital Medicine, Ochsner Clinic Foundation, and The University of Queensland Faculty of Medicine, Ochsner Clinical School, New Orleans, LA

## SUICIDE AMONG MEDICAL PROFESSIONALS

The suicide rate among male physicians is 1.41 times higher than among the general male population. Among female physicians, the relative risk is even more pronounced—2.27 times greater than the general female population.^1^ I personally knew 4 physicians and 1 advanced practice provider who committed suicide. The causes of suicide are complex and include feelings of being overwhelmed, grief, isolation, lack of social support, discrimination, intolerable emotional pain, stress, and feeling trapped and hopeless.^2^ In psychological terms, suicide represents the buildup of uncontrolled toxic negative thinking, while gratitude represents the opposite end of the spectrum toward positivity.

## PREVENTIVE EFFECTS OF GRATITUDE

Gratitude, the “tendency to recognize and respond with grateful emotion to the roles of other people's benevolence in the positive experiences and outcomes that one obtains,”^3^ is negatively correlated to suicidal thoughts and suicide attempts.^4-6^

No direct evidence shows that gratitude prevents suicide in health care providers, but having said that, I believe that it does. Research is needed. The study of the effect of gratitude on well-being is in its infancy at less than 20 years.^4^ Krysinska conducted a review of the evidence base for gratitude as a protective factor for suicidal ideation.^4^ She reported on 12 studies that link gratitude with reductions in suicide and protective effects against suicide among children and adolescents, college students, and veterans.^4^ Studies of adolescents and children confirm gratitude's influence on reducing suicidal behaviors and ideation and the benefits of gratitude in suicide prevention.^4^ Research has found a weaker association between suicidal ideation, hopelessness, and depressive symptoms in grateful individuals compared to ungrateful individuals.^4,5^ Strong evidence shows that gratitude is related to psychosocial and physical well-being.^4,5^ Gratitude may have preventive effects against depression, anxiety, and posttraumatic stress disorder. Gratitude is known to boost interpersonal relationships, health, general emotional functioning, and self-development. It improves sleep and somatic symptoms. Furthermore, the relationship between gratitude and suicidality has been shown to be mediated by self-esteem**.**^7^ There is a direct association between higher levels of gratitude and higher self-esteem. In girls who have been victims of bullying, gratitude has been shown to buffer the relationship between victimization and suicide risk.^8^ Research has also determined that when an individual has fewer life stressors, gratitude has more potent effects to prevent suicide.^3^ Therefore, gratitude interventions in times of less stress may be important to induce a resiliency factor for suicide prevention.

Gratitude's protective role is enabled by generating meaning, belonging, hope, appreciation, and positivity. World War II concentration camp survivor, Dr Viktor Frankl wrote, “The impulse for suicide could be overcome if one can find meaning in one's life.”^9^ Gratitude is known to be a buffer against the risk factors of hopelessness and depressive symptoms, which often lead to suicidal ideation and behavior. Distinguished from future-focused emotions such as optimism and hope, feelings of gratitude are focused on the present moment without expectation for the future. This fact may be a significant influence on the prevention of suicide by improving impulse control. People who can feel gratitude regularly are more satisfied with their lives. Also, when higher levels of gratitude are present, the more likely someone is to come to the aid of another. The practice of gratitude has the ability to produce more positive emotions, more rewarding social interactions, and improved social support. It also can reduce negative moods. Self-critical individuals are particularly responsive to the benefits of gratitude intervention. Gratitude may be able to counter the negative aspects of perfectionism learned in medical school and potentiated by an active and stressful medical career.

## PSYCHOLOGICAL CONCEPTIONS OF GRATITUDE

There are 3 psychological conceptions of gratitude.

1. The first is a moral affect.^4^ Gratitude is an emotion associated with the felt experience of awe. People (beneficiaries) respond with gratitude when other people (benefactors) behave in a way that promotes the beneficiaries’ well-being. Beneficiaries also act in ways that promote the well-being of others when they themselves have been made grateful. Gratitude functions as a barometer, a motivator, and a reinforcer. People are likely to experience gratitude when they perceive the generosity of a benefactor. Then, gratitude inspires beneficiaries to behave prosocially. And finally, a beneficiary's expression of gratitude is likely to motivate (ie, morally reinforce) a benefactor to engage in prosocial behavior in the future.
2. The second conception of gratitude is as an affective trait, an aspect of personality.^4^ A trait describes a quality or characteristic that typically belongs to a person. A person with a gratitude trait has a generalized tendency to recognize and respond with grateful emotion to the role of other peoples’ benevolence in positive experiences and outcomes. Character traits can be further described by their intensity, frequency, and density.^4^ They can be developed over time.^6^
3. The third conception is a life orientation.^4^ Gratitude becomes part of a wider life orientation toward noticing and appreciating the positive in the world. A life filled with gratitude evokes appreciation, understanding of individual differences, awe, focus on what a person has, being in the present moment, understanding the impermanence of life, and making positive social comparisons.^4^

There are many hypotheses for the positive effects of gratitude, and they are likely interlinked.^10^ The coping hypothesis holds that grateful people cope in positive ways with beneficial social support and personal resourcefulness, and they avoid harmful coping methods such as alcohol and drugs.^4,10^ In the schematic hypothesis, gratitude is evoked by having received help that is appraised as costly to provide, valuable, and altruistically offered, rendering in the grateful person positive interpretive bias, such that the person makes benevolent appraisals. The beneficiary becomes more likely to harvest the personal and interpersonal benefits of gratitude.^4,10^ The broadening and building hypothesis describes how experiences of gratitude draw attention to the positive aspects of the social environment, allowing people to discover novel ways of perceiving the world and solving problems.^4,10^ They see the world in a positive light. The individual can build a repository of effective coping skills, social support, and other intrapersonal and interpersonal skills that help them deal with life's problems and enhance life satisfaction that, in turn, can result in experiencing more positive experiences and emotions, maintaining an upward spiral toward enhanced well-being. Last is the positive affect hypothesis.^4,10^ The positive neurologic effects when gratitude becomes a habit are protective against psychosocial ill-being and contribute to life satisfaction. Gratitude enhances the ability to recall positive memories. Gratitude reduces envy and resentment (negative emotions that result from social comparisons) and reduces regret (negative emotions that result from self-comparison). Gratitude reduces materialistic striving and is positively related to self-esteem and self-respect.^4^

Gratitude is a character strength of the virtue of transcendence.^6^ It is linked to positive words such as kindness, friendship, happiness, love, harmony, help, thanksgiving, satisfaction, fulfilled, deserving, meaning, appreciation, connectedness, worthiness, care, wholeness, purpose, consideration, good, and many more. When we are grateful, all these other positive cognitions are stimulated, develop internally, and grow. Gratitude develops its positive effects gradually.^11^ Words matter. How we train ourselves to feel matters. What we put into our brains eventually comes back out. We externalize these words and feelings with our speech and actions, and they transform our surroundings and us.^12,13^

## CULTIVATING GRATITUDE

Gratitude is a personal resource that we can readily access and cultivate. Gratitude can be measured by the Gratitude Questionnaire-6.^3^ This survey is an eye-opening 6-item form that is easily found online. It measures peoples’ experience of gratitude as an emotion or affect. The scores range from 6 to 42, with the higher numbers correlated with higher gratitude disposition. Scores less than 38 are below the 50th percentile. The Gratitude Resentment and Appreciation Test is a more extensive 44-item self-report that measures 3 different subscales: lack of a sense of deprivation, appreciation of simple pleasures, and social appreciation. By understanding your disposition, you can apply your attention to the appropriate subscale in your meditation or gratitude practice.^4^

Awareness of the positive effects of gratitude should be made mainstream. Gratitude ought to be taught and intentionally practiced by everyone in a group to increase positive experiences in general. The practice of gratitude holds the promise to be a low-cost, high-impact tool that enhances existing suicide interventions.^14^ Appreciation of others and recognition of each other's character strengths and good works are team-building exercises that promote the well-being of all individuals in a group. In a high-stress work environment such as hospital medicine, intentional efforts to promote a positive influence on well-being need to be undertaken by everybody on the team to establish a proper equanimity of mental functioning.

Intentional group practice of gratitude can set up a baseline boundary of the expectation of respect for every individual. The cumulative effect over time is to cultivate a positive prosocial environment that precedes the development of hopelessness and depressive symptoms, reciprocates positivity, and protects the mental health of hospitalists from any negativity that is present. A medical group practice that intentionally cultivates gratitude and a positive work environment is a practice that puts death, dying, and the human condition into the proper perspective of a greater positive humanity.

For the individual provider, expressing gratitude can feel awkward when negative cognitions are present. Providers who express and feel gratitude activate the limbic system and the learning reward system, release toxic emotions, and increase the volume of grey matter in the right inferior temporal gyrus.^11^ Practicing gratitude builds the mental muscles that show up as positive emotions that affect speech and behavior. The habit of expressing gratitude leads to the felt experience of gratitude. Gratitude becomes a lifestyle. But just being grateful is not enough. To receive the social benefits of gratitude and open the world to new possibilities, we must express gratitude publicly.^15^According to the American philosopher Adam Smith, gratitude opens the doors of reciprocity, prompts humans to reward each other, and binds people together in a society. Gratitude drives social equilibrium.^15^

Thankfulness is an action that humans take after receiving a gift. Thankfulness triggers reciprocal benefits and maintains a relationship. Gratitude is deeper and emotional; it acknowledges dependence and elicits humility. Gratitude calls forth an openness and engagement with the broader community.^15^

Gratitude is a social-emotional response that signifies well-being.^11^ A positive work environment recognizes the empathy-compassion dynamic and the trauma response at work between providers and patients. Although gratitude is positively associated with wellness, that wellness does not emanate from leaders who have gratitude for their frontline providers. Wellness arises from frontline providers who experience the felt emotion of gratitude for their organizations. That subjective felt experience is one of awe, wonder, love, and fulfilment of hope. The felt experience of gratitude signifies a well-functioning somatic sensory nervous system and ventral vagus nerve, proper shutdown of the dorsal vagus nerve and the trauma response, and integration of the insula with the mirror neuron system of the emotional brain.^16^ Proper functioning of the somatic sensory system enables neurosynchrony, attunement, and attachment, all of which are needed for the right operation of a team, the perception of gratitude, and, likely, suicide prevention. The healing power of gratitude is generated when the emotional memory of the felt gratitude links with a biographical memory of a positive experience by maintaining the longevity of that memory. The felt experience of gratitude on the front line brings a greater goodness to the organization, the patients, and the providers.

The impact of COVID-19 on physician mental health and suicide is not known. Media attention has been paid to the concern for increased rates of physician suicide because of increased work demands, social isolation, decreased self-care, and increased exposure to emotionally traumatic events at work and home.^17^ While organizations need to cultivate the culture with the conditions that ripen gratitude experience on the front line, providers need to combat the negativity bias inherent in the practice of medicine. The practice of gratitude is an entryway to positive psychology. The intentional practice of gratitude opens the door to true healing that comes from loving-kindness, forgiveness, and self-compassion.

From time immemorial, physicians have been on the front lines during wars, disasters, and pandemics, tending to the struggles of humanity. The selfless acts of compassion that hospital providers perform often go unnoticed by the people who are suffering, and providers are witnesses to the humanity and the inhumanity that accompany life. Despite these challenges, the practice of medicine is a joy and a privilege. Physicians must take care of themselves by communicating boundaries and engaging in self-care to complete their life's work.^18^ Start by repeating the mantra, “I am grateful for my life.”
